# Diagnostic and therapeutic approach to abdominal masses in a country with limited resources

**DOI:** 10.1186/s12893-024-02371-w

**Published:** 2024-03-23

**Authors:** Saïdou Adama, Mohamed Lamine Abani Aichatou Balaraba, Zabeirou Oudou Aliou, Bako Inoussa Daouda, Ide Kadi, Younssa Hama, James Didier Lassey, Sani Rachid

**Affiliations:** 1 Department of Surgery and Surgical Specialties, Reference General Hospital, BP 12674, Niamey, Niger; 2 Medical Technical Support Department, Reference General Hospital, BP 12674 Niamey, Niger; 3 Department of Surgery and Surgical Specialties, National Hospital BP 238, Niamey, Niger; 4 Department of General and Digestive Surgery, Amirou Boubacar Diallo Hospital, BP 10146, Niamey, Niger

**Keywords:** Abdominal Masses, Imaging, Laparoscopy, Niamey

## Abstract

**Objective:**

To report the diagnostic and therapeutic approach for the management of abdominal masses in the General Surgery department of the Niamey General Reference Hospital (HGR).

**Materials and methods:**

This were a retrospective and preliminary study of 2 years and 3 months on patients operated for abdominal masses in the General Surgery department of the HGR. A palpable mass and/or its size on imaging (40 mm) were the inclusion criteria.

**Results:**

Abdominal masses accounted for 6.7% (*n* = 53) of other pathologies. The average age of the patients was 41.26 years, with a standard deviation of 14.2 and a female predominance of 75.5% (*n* = 40) with a sex ratio of 0.32. The abdominal mass was clinically palpable in 75.5% (*n* = 40). Abdominal pelvic ultrasound was performed as a first step in all patients and in 75.5% (*n* = 40) it specified the origin of the mass. Contrast-enhanced abdomino-pelvic CT scan, performed in 52.8% of patients (*n* = 28) and in 89.3% (*n* = 25) specified the preoperative diagnosis. The most frequent etiologies were uterine fibroids, 35.8% (*n* = 19). In 5.6% (*n* = 3) the diagnosis was not precise preoperatively despite the two imaging studies, and these patients had underwent exploratory laparotomy. Surgery was the initial therapeutic approach for all patients, and laparoscopy accounted for 22.6% (*n* = 12). Postoperative complications occurred in 7.5% (*n* = 11). The death rate was 5.6% of cases (*n* = 3).

**Conclusion:**

Imaging remains important in the etiological research for abdominal masses. Definitive treatment remains surgical; mortality would be linked to the malignant nature and the significant volume of the mass.

## Introduction

Abdominal masses correspond to an increase in the volume of an organ or region of the abdomen; they include all the intra-abdominal masses and the antero lateral wall of the abdomen. It may be benign, malignant, malformative or infectious [1]. An abdominal mass can be detected through a thorough physical examination, though sometimes its discovery is incidental during imaging exam [1]. Clinical manifestations are variable depending on location, mass size, and can sometimes be asymptomatic or result in complications. Imaging, endoscopy, and biology play an important role in the diagnostic process, as well as surgery that contributes to both diagnosis and treatment [2]. It is essential to distinguish between the masses to be treated immediately [1]. The etiological diagnosis depends on the age of the patient and the site of the mass; nevertheless, it is always the histological examination that gives a diagnosis of certainty [2, 3]. The treatment is primarily surgical, the modalities of this surgery depend on the pathology, its extension, and its topography. What is the contribution of imaging in the diagnosis of these abdominal masses? The objective of this study is to report the experience of the Reference General Hospital (RGH) in the diagnostic and therapeutic approach of abdominal masses with limited means in the department of general and digestive surgery.

## Materials and methods

This was a preliminary, prospective and retrospective, descriptive and analytical study conducted over 2 years and 3 months (from January 1, 2018, to March 31, 2020) in the General and Digestive Surgery department of the Niamey General Reference Hospital (HGR); a new tertiary level hospital opened on November 26, 2017, to serve as a reference for all other hospitals in Niger. The study population included all patients treated for an abdominal mass in the department. After the clinical examination, ultrasound (Mindray DC 70, put into service in 2019) was the first imaging test requested, followed by CT scan (Neusoft, 16 Barrettes, put into service in 2016) in second position if needed. Sometimes, both ultrasound and CT scan were necessary for the same patient. CT scan was systematically performed with contrast injection. MRI was not available in the hospital at the time of the study. Included were patients of all ages with an abdominal mass, whether peritoneal, retroperitoneal, abdomino-pelvic, or developed in the abdominal wall, clinically palpable, and/or measuring a size greater than or equal to 40 mm on imaging, operated on an emergency basis or in scheduled surgery. The parameters studied were epidemiological, clinical, paraclinical, diagnostic, therapeutic, and evolutionary aspects.

## Results

**Fig. 1:**
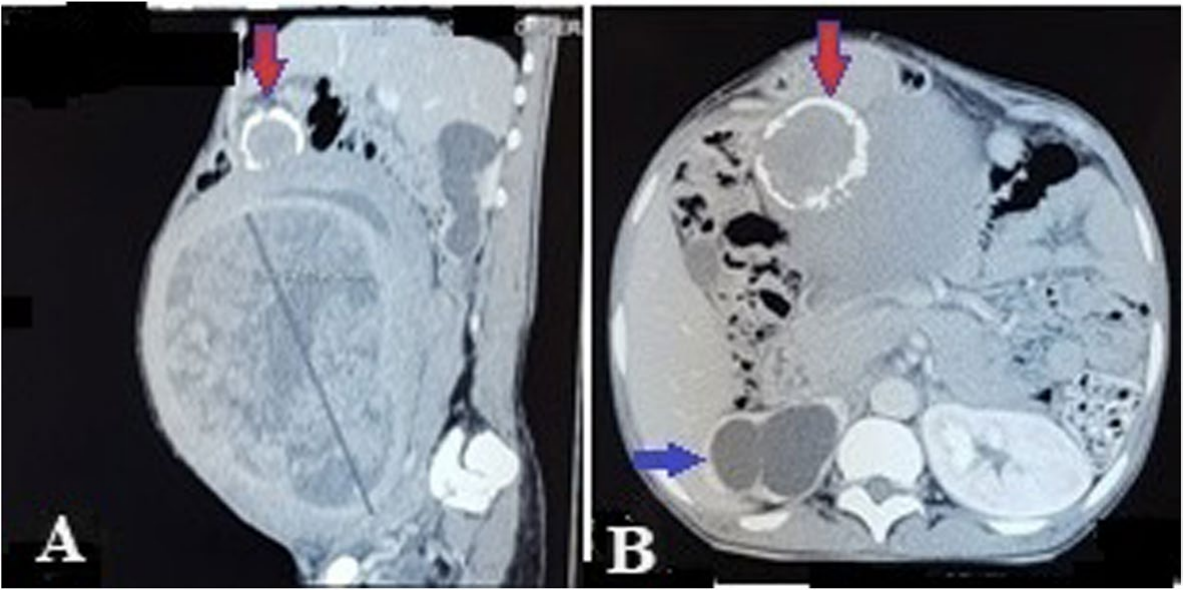
CT abdomino pelvis injected with a large mass abdominal cavity: frontal section (**A**) Poly nodular fluid tissue mixed tumor process occupying virtually the entire abdominal cavity, with thick and even wall taking contrast; a calcified tissue nodule (red arrow). The measurements were 210,64 × 172,69 × 229 mm (**B**) axial section: The lesion exerts a mass effect on the urinary tract and the bladder giving a right hydronephrosis uretero, pyelic diameter 18, 89 mm (blue arrow)

**Fig. 2:**
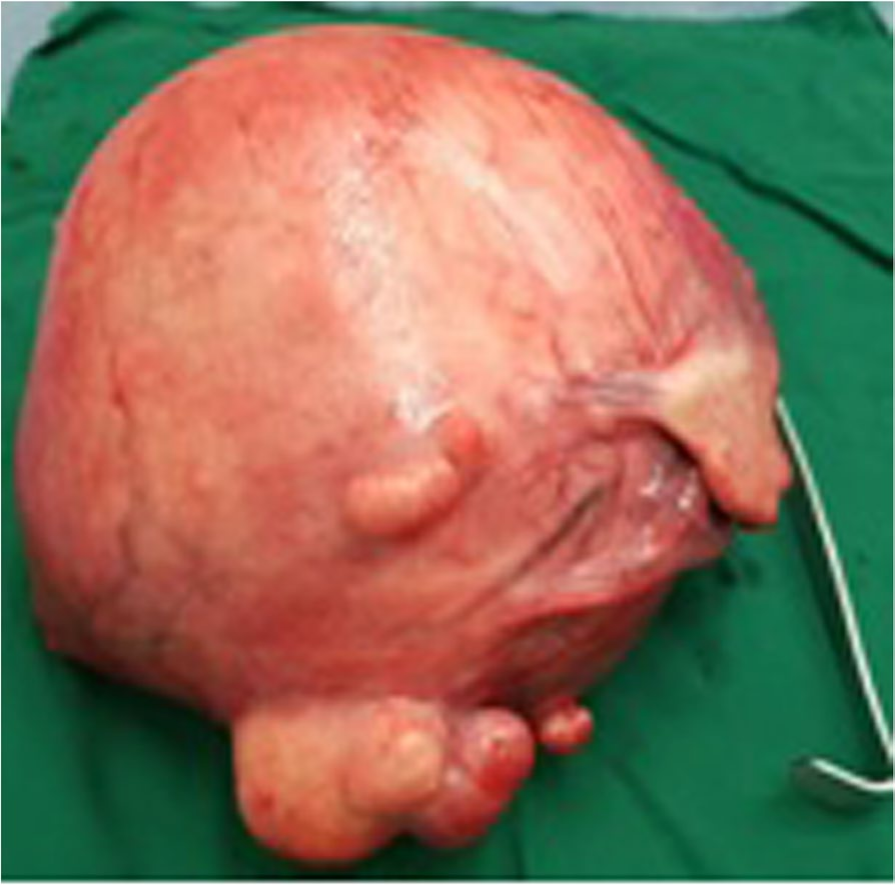
Operative part of Fig. 1: total hysterectomy part and unilateral annexectomy

**Fig. 3:**
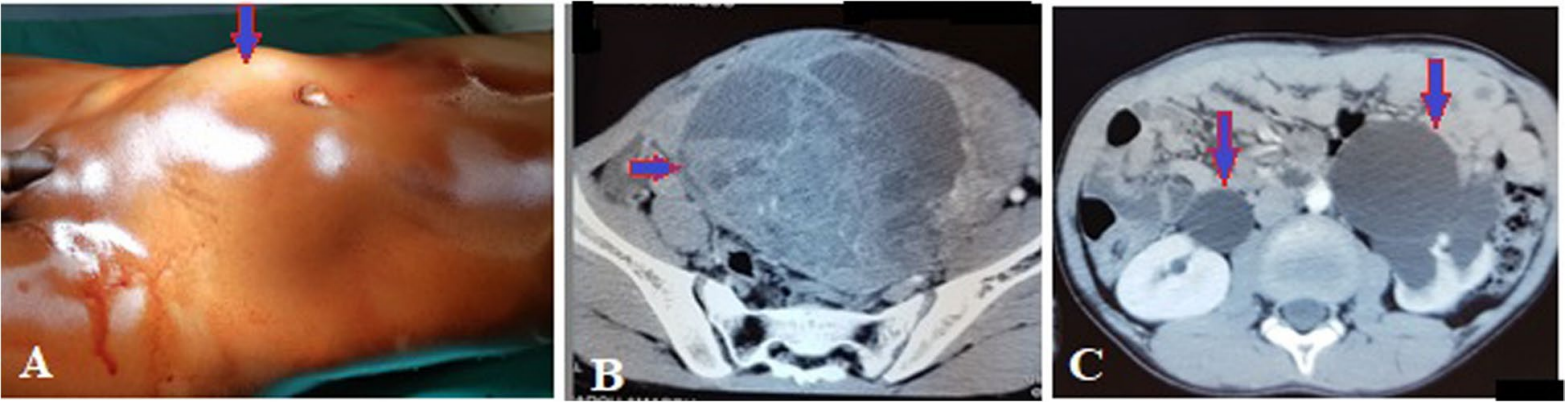
Pelvic mass in a 12-year-old child (**A**): represented by the axial slice CT image (**B**) of an irregular pelvic mass with tissue and fluid content, resulting in a bilateral compression hydronephrosis uretero (**C** blue fleches)

**Fig. 4:**
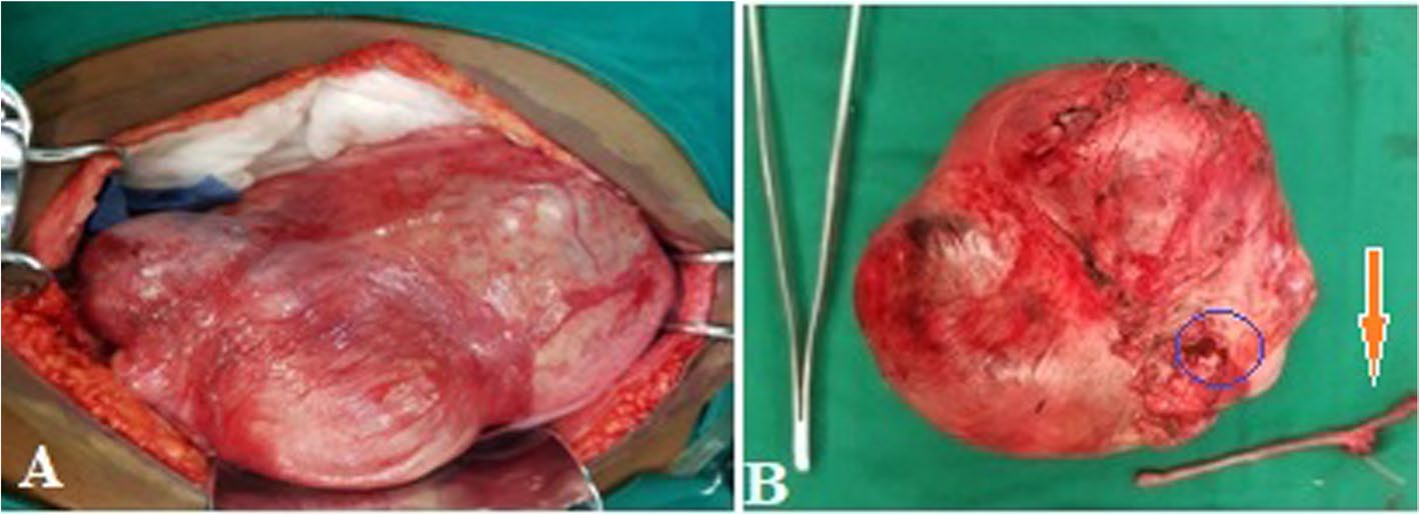
Per operative images of young 12-year-old patient: Per-operative view of a pelvic mass in a 12-year-old child (**A**); A residual resection “R1” by tumor break-in and left ureteral invasion. Secondary ureteral resection (**B**) and bladder re-installation was performed

**Fig. 5:**
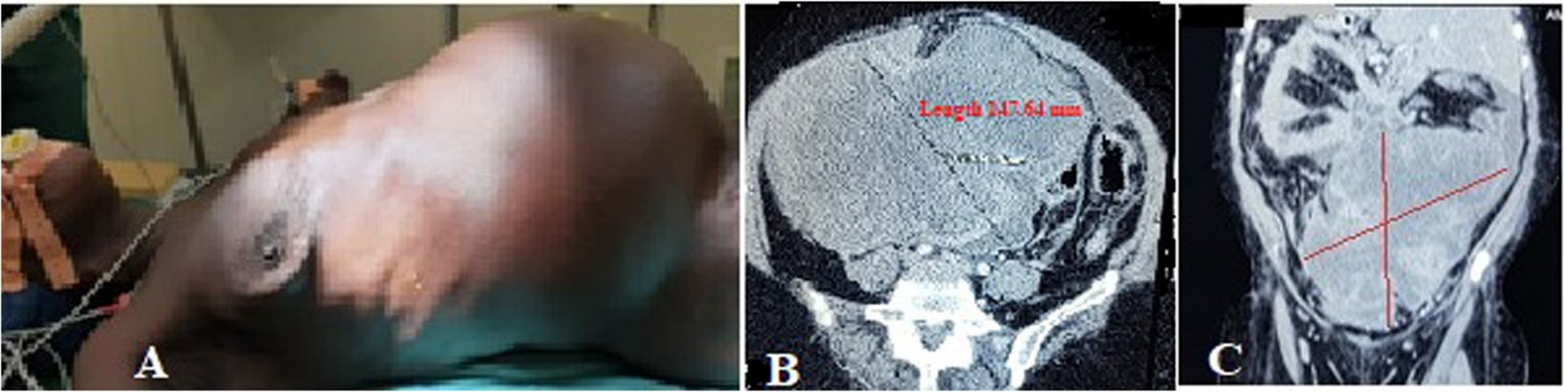
Pelvic mass installation (**A**) in a 58-year-old patient; axial section of an irregular pelvic mass with fluid tissue content (**B**), sagittal section, it goes up in contact with the mesenteric tumor (**C**)

Abdominal masses accounted for 6.7% (*n* = 53) among 790 patients treated in the General and Digestive Surgery department during the same period. The female sex was predominant with 75.5% (*n* = 40), giving a sex ratio of 0.32. The average age was 41.8 years ± 14.2 years, including one patient under 15 years of age. The age group of 30 to 45 years was the most represented with 41.5% (*n* = 22). Patients with a body mass index (BMI) higher than kg/m² represented 17% (*n* = 9). Abdominal pain was the reason for consultation reported by 84.9% of patients (*n* = 45). The mass was clinically palpable in 75.5% of patients (*n* = 40), and exclusively pelvic location accounted for 32% (*n* = 17). The mass was hard and irregular in consistency in 47.5% of cases (*n* = 19), fixed to the deep plane in 60% (*n* = 24), and tender in 62.5% of patients (*n* = 25). Abdomino-pelvic ultrasound was performed in all patients, and in 75.5% (*n* = 40) it specified the origin of the mass by providing a contributory diagnosis that was confirmed perioperatively. It allowed for guided puncture in 4 cases (7.5%). Contrast-enhanced abdomino-pelvic CT scan, performed in 52.8% of patients (*n* = 28); it was contributory, and the diagnosis was consistent perioperatively in 89.3% (*n* = 25). In these imaging studies, solid masses were the most found in 70% of cases (*n* = 37), followed by cystic masses in 26.3% of cases (*n* = 14) and mixed masses in 3.7% of cases (*n* = 2). Retroperitoneal masses accounted for 13.2% (*n* = 7) and abdomino-pelvic masses 86.8% (*n* = 46). The most represented preoperative diagnoses were uterine fibroids in 35.8% (*n* = 19), ovarian cysts in 11.3% (*n* = 6), and ovarian tumors in 9.4% (*n* = 5). Ultrasound was more precise, and the diagnosis was concordant preoperatively in uterine fibroids, ovarian cysts, and renal and adrenal tumors, splenomegaly, hydronephrosis; however, at ultrasound, the diagnosis was less precise in cases of tumor of the pancreas tail, gastric tumor, and of the caecum (represented in blue in Table 1) and these diagnoses were made by contrast-enhanced abdomino-pelvic CT scan performed in second position. It was necessary to couple ultrasound with CT scan in 47.17% (*n* = 25) of cases. When combining both imaging modalities, the origin of the mass was not well specified in 5.6% (*n* = 3) of cases, and exploratory laparotomy was indicated (Table 2): The first case involved a 32-year-old nulligest patient with an abdomino-pelvic mass. A contrast-enhanced abdomino-pelvic CT scan was performed (Fig. 1) after a non-contributory ultrasound, and the diagnosis remained uncertain between an ovarian tumor or a uterine tumor. Per operative, it was a polymyomated uterus necrobiosis with calcifications occupying the entire abdominal pelvic cavity. A total hysterectomy with unilateral annexectomy was performed (Fig. 2). The second case involved a 12-year-old child (Fig. 3a) presenting with a pelvic mass. The contrast-enhanced abdomino-pelvic CT scan showed a budding pelvic mass with tissue-liquid content (Fig. 3b) with compression of the urinary tract resulting in bilateral ureterohydronephrosis (Fig. 3c). An exploratory laparotomy was performed (Fig. 4a) and a complete resection of the tumor was carried out (Fig. 4b), complicated by tumor rupture (blue circle) and invasion of the left ureter (resected and re-implanted into the bladder over a JJ stent); The specimen of the resected ureter in the image (Fig. 4b, orange arrow). The histological examination of the surgical specimen confirmed an intra-abdominal soft tissue sarcoma, R1 resection. The third patient, a 58-year-old, presented with an abdominal mass and a contrast-enhanced abdominal CT scan had been performed, suggesting a mass of mesenteric origin (Fig. 5a), and an injected abdominal CT was performed, suggesting a mesenteric mass (Fig. 5 b and c). The intraoperative diagnosis was an unresectable abdominal tumor with peritoneal carcinomatosis; a biopsy was performed confirming an ovarian carcinomatous tumor with peritoneal carcinosis. In addition to etiological diagnosis, the CT scan was performed for a staging assessment for digestive tumors such as colorectal, gastric, adrenal, renal, and pancreatic tumors. Neoadjuvant chemotherapy was administered in 5.6% of patients (*n* = 3), involving one case each of left renal tumor, head of pancreas tumor, and right colon tumor. General anesthesia was performed in 96.2% of patients (*n* = 51). The approach was laparotomy in 69.8% of cases (*n* = 37), laparoscopy (Table 3) in 22.6% of cases (*n* = 12), and lumbotomy in 7.6% (*n* = 4). The most frequently performed surgical procedures were myomectomy, hysterectomy, and adnexectomy in 22.6%, 18.9%, and 17% of cases, respectively. The average duration of hospital stay was 9.5 ± 4.3 days, with a range of 3 to 21 days. For patients operated on laparoscopically, the average hospitalization time was 2.3 days. The histological nature was known in 56.6% of patients (*n* = 30). There were 13.2% (*n* = 7) malignant tumors, 43.4% benign tumors (*n* = 23) including uterine leiomyoma, which accounted for 20.7% of cases (*n* = 11). Adjuvant chemotherapy was administered in 9.4% of patients (*n* = 5). These were two cases of gastric tumor, two cases of right colon tumor and soft tissue sarcoma. Early postoperative outcomes were simple in 79.3% of cases (*n* = 42) and complicated in 20.7% of cases (*n* = 11), of which 7.5% (*n* = 4) were classified grade IIIb according to the Clavien-Dindo classification. The long-term outcome was favorable in 94.4% of cases (*n* = 50) and marked by 5.6% (*n* = 3) of deaths, including one case of right colon tumor who died from a complication of adjuvant chemotherapy, one case of head of pancreas tumor who died from liver metastases, and the child with sarcoma who died in a picture of multivisceral recurrence.

**Table 1:** Ultrasound evoked pre-operative diagnostics

	Localization	Diagnosis	Effective	Percentage%
		Uterine myomas	18	33,9
		Ovarian cyst	6	11,3
		Ovarian tumor	5	9,4
		Ovarian tumor ?	1	1,9
		Tropical spleen	1	1,9
	Intra péritonéale	Liver abscess	4	7,5
		Right colon tumor	4	7,5
Échographie		Gastric tumor	2	3,8
		Caecum tumor	1	1,9
		Appendicular abscess	1	1,9
		Mesenteric cyst	1	1,9
		Mesenteric tumor	1	1,9
	Retro péritonéale	Pelvic tumor	1	1,9
		Kidney tumor	3	5,7
		Pancreatic tail cyst	2	3,8
		Left hydronephrosis	1	1,9
		Left adrenal tumor	1	1,9
Total			53	100

**Table 2:** Patients in whom the ultrasound coupled to the scanner, the diagnosis was not accurate

Diagnosis	Radiological diagnostics	Per-operative diagnostics	Context or review allowed diagnosis
Imageries			
	Ovarian tumor? uterine?	Géant myome utérin	Per operative, histology
Ultrasound + CT	Pelvic tumor ?	Pelvic mass	Histology: Sarcoma tissue
			Soft tissue
	Is that a mesenteric tumor?	Abdominal tumor +	Histology:
		Peritoneal carcinosis	Ovarian carcinoma

**Table 3:** Indications of laparoscopy

Laparoscopic indication	Laparoscopic gestures	Effective	Percentage %
Liver abscess	Drainage, washing	3	25
Mesenteric cyst	Resection	2	16,6
Bilateral ovary cysts	Resection	2	16,6
Right ovarian tumor	Right ovariectomy	1	8,3
Right colon tumor	Right hemicolectomy	1	8,3
Appendicular abscess	Appendectomy, washing	1	8,3
Pancreatic cyst	Drainage	1	8,3
Adrenal tumor	Adrenalectomy	1	8,3
Total		12	100

## Discussion

Abdominal masses are a common reason for surgical consultation. They hide behind several diseases, both benign and malignant. We report a study on abdominal masses, regardless of the etiology and age group in a new hospital, of reference in Niger, which has not fully completed the installation of its imaging devices. We report a frequency of 6.7% of abdominal masses among all the activities of the digestive surgery service of the HGR during the study period. Our results are higher than those reported by Okoko A-R. et al. in Brazzaville in 2012 [4]. who focused solely on children, with a frequency of 1.3%. The study showed a female predominance of 75.5% with a sex ratio of 0.32. This female predominance is consistent with findings by Akkoca M. et al. [5]. in Turkey in 2017 (60%) and Mahamoud G. et al. [6] in Morocco in 2010 (54.1%). The average age of our patients was 41.8 years ± 14.2, with a range from 10 to 69 years. Few studies have reported on all ages as ours did; literature typically focuses on studies involving children; Children’s studies are generally found in the literature [7]. The prevalence of abdominal masses by age is not widely discussed. However, neoplastic abdominal masses are rare in the first year of life but become very common between ages 1 and 6 [4]. Abdominal pain was the main symptom in most of our patients (84.9%). Lower rates were found by Akkoca M. et al. and Mahamoud G. et al., at 61% and 57.5% respectively [5, 6]. In younger patients, abdominal masses are often incidentally discovered by a parent [7].

In our study, a palpable abdominal mass was found in 75.5% of cases, even though 17% of patients were obese. The mass was hard and irregular in 47.5% of cases, tender in 62.5% of cases, and fixed in relation to the deep plane in 60% of cases. Akkoca M. et al. [5], in a series of 43 cases in Turkey in 2017, found abdominal masses on physical examination in 62.2% of cases. This difference in frequency could be because our study only focused on large abdominal masses, thus making them more accessible for examination.

Abdomino-pelvic ultrasound is currently the reference technique for diagnosing abdominal masses. It allows for topographical diagnosis of the mass, attachment to an organ, precise determination of the tumoral volume, and definition of its solid or liquid nature [8]. In an American literature review published in 2019, authors affirmed an accuracy of 88 to 91% for ultrasound in determining the organ of origin and 77 to 81% in diagnosing the underlying pathology [9]. In our study, ultrasound was performed in 100% of cases. It specified the site of the mass and the organ of origin in 75.47% of cases (uterine myomas, ovarian cysts, renal tumors…) and contributed to the performance of echo-guided biopsy punctures in 4 cases. Our results are similar to those of Kathryn J.F. et al. [9] in the USA in 2019, who reported ultrasound performance rates of 100% in their series. In case of clinical suspicion of abdominal mass, the ultrasound requested in 1st intension is justified (strong professional agreement), and it is a first-line examination requested in the literature, it is less expensive, non-invasive and very well supported by the patient [8]. However, its performance can decrease because it is an operator-dependent examination. In the context of abdominal emergencies, the sensitivity of ultrasound is often significantly lower than that of CT, which is preferred in most indications [10]. Furthermore, as one author highlighted, "the effectiveness of ultrasound depends on the relevance of the request," [2]. meaning that the more clinical information is accurately reported, the better it can guide the operator. Abdominopelvic CT is the key examination in the assessment of abdominal masses. It provides valuable information for localizing the mass by anatomical compartments, organ localization, characterization, and assessing the extent of these masses [2]. Over the last 15 years, there has been a rapid technological evolution from sequential to spiral, single to multidetector modes. This evolution currently allows for investigating an abdomen in a few seconds with spatial resolution less than 1 mm, enabling routine multiplanar reconstructions of equal quality to the initial acquisitions [10]. In our series, CT was performed in 52.8% of cases and contributed to the etiological diagnosis in 89.29% of cases. Our results are lower than those found by Akkoca M. et al. [5], in Turkey in 2017, who reported 62.2%. The lower performance of CT scan, compared to ultrasound, could be explained by the high cost of this examination, especially in our context where most patients do not have social security, and because it is not indicated as a first-line examination, especially in young patients due to its irradiating nature (often requiring the injection of iodinated contrast agent). Limited access to modern diagnostic tools, due to their high cost and the lack of social security for patients, makes the hospital the last resort in case of illness. These factors lead to delays in patient care and result in some tumors, especially malignant ones, being seen late and at an advanced stage. We emphasize the physical examination of patients, which guides the diagnosis. It plays a significant role in diagnosing abdominal masses, especially palpable ones. The various topographical and semiological characteristics of these palpable masses during the examination, accompanying signs (such as pain, metrorrhagia, rectal bleeding), location, regular or irregular nature, progression, and the general state of the patient, guide the surgeon towards a diagnosis. However, currently, imaging is recommended for any abdominal mass [8]. Despite performing ultrasound and CT scan, there were three cases where the diagnosis was not precise. The diagnosis of this intra-abdominal soft tissue sarcoma could not be established pre-operative by CT in our study. MRI remains the reference exam for the local Soft Tissue Sarcoma because it has excellent tissue contrast [11]. The tumor was compressing both ureters and the possibility of a neoadjuvant treatment such as radiotherapy in Niger was almost impossible at a time when the country did not have it. It was necessary to request a medical evacuation outside that would have lasted several months. Exploratory laparotomy was indicated and R0 resection could not be obtained due to tumor invasion of the left ureter. Despite the adjuvant chemotherapy, the recurrence had been very rapid and overwhelming. The 2nd case involved a tumor occupying the entire abdominal cavity, and it is reported that the scanner is ineffective in determining the origin of the organ in these cases of giant tumor of the abdomen (interest of multiplanar reconstruction) [2]. And per operation, it was a large poly myomatous uterus and necropolis. The last case concerned a suspicion of a mesenteric tumor at the injected scanner and the discovery of a peritoneal carcinosis from an ovarian tumor per operative was accidental. The diagnosis of peritoneal carcinomatosis is easy in diffuse and macronodular forms with ascites. Rough shapes are difficult to diagnose. Imaging peritoneal carcinoses [12] remains first and foremost a technical challenge in radiology: because it requires high spatial resolution (especially in small lesions) and high contrast resolution (nodules have low spontaneous contrast with no significant enhancement) and finally there is a minimization of motion artifacts due to the contact of the handles that are moving. Secondly, it is a radiological challenge because peritoneal carcinoses have low reproducibility, and most are seen only in surgery. However, the scanner is still the reference tool, but it has limited sensitivity per organ site, especially at the pelvic level [13]. The FDG-PET scan and/or diffusion MRI is recommended [12]. However, our hospital did not have an MRI at the time of the study and the entire country does not have a Pet Scan. This notes the limitations of our study, where some series reported the diagnostic effectiveness of radioguided puncture, today, despite its invasive nature, it enters the diagnostic approach of abdominal masses [14, 15].

In our study, we found a significantly higher frequency of uterine fibroids (35.8%), followed by ovarian cysts in 11.3% of cases and ovarian tumors in 9.7% of cases. This could be related to the female predominance (75.5%) and the young average age (41 years) of our study population. Thus, as reported by most authors, uterine fibroids and ovarian cysts represent the main etiologies of abdomino-pelvic masses in black women during their reproductive and pre-menopausal periods [13, 16]. The choice of therapeutic approach depends on the histological type, the extent of the mass, and the patient's age [17]. For our patients, two treatment methods were used: chemotherapy and surgery, as radiotherapy was not yet available in Niger. These methods were in some cases well indicated and in others adapted to the work context.

Surgery was the main therapeutic recourse (100%). We reported an exceptional case of total hysterectomy in a 32-year-old nulliparous woman carrying a large necrotic uterine fibroid that had occupied almost the entire abdominal cavity. It was responsible for severe anemia with vomiting (due to gastric compression). The hysterectomy specimen (preserving an ovary) measured 29 mm x 23 mm in height.

In our series, the outcome was favorable for the majority of patients. Nevertheless, a mortality rate of 5.6% was reported for all abdominal masses. All deceased patients had a malignant tumor. Most of these patients had consulted at an advanced stage of their diseases. Our results are higher than those found by Akkoca M et al. [5], who reported a mortality rate of 4.4%.

## Conclusion

Abdominal masses hold a significant place in the activities of the General and Digestive Surgery Department of the HGR. Imaging plays a crucial role in the investigation and etiological orientation of abdominal masses. It allowed for the preoperative diagnosis in the majority of cases. Ultrasound was the examination of choice requested as a first step, followed by CT. Surgery remains sometimes our only diagnostic and therapeutic option due to the lack of a more advanced technical platform. The outcome seems favorable in the majority of cases; however, mortality is linked not to diagnostic failure but to the malignant nature, the extent of the disease, and the large volume of the mass.

## Data Availability

All data generated or analysed during this study are included in this article.

## Abbreviations

RGH: Reference General Hospital
BMI: Body mass index
MRI: Magnetic resonance imaging
CT scan: Computerized tomography *scan*
FDG-PET: F-fluorodeoxyglucose-positron emission tomography
